# Experiences with screening for atrial fibrillation: a qualitative study in general practice

**DOI:** 10.3399/BJGPO.2021.0126

**Published:** 2022-02-23

**Authors:** Steven B Uittenbogaart, Stéphanie JE Becker, Maartje Hoogsteyns, Henk CPM van Weert, Wim AM Lucassen

**Affiliations:** 1 Amsterdam University Medical Centers, University of Amsterdam Department of General Practice, Amsterdam, The Netherlands

**Keywords:** general practice, atrial fibrillation, diagnostic techniques, cardiovascular, pulse, electrocardiography, qualitative research

## Abstract

**Background:**

Guidelines recommend screening for atrial fibrillation (AF). Currently, screening is not considered standard care among GPs.

**Aim:**

To explore the experiences of primary care workers with different methods of screening for AF and with implementation in daily practice.

**Design & setting:**

A qualitative study using semi-structured interviews with GPs, nurses, and healthcare assistants (HCAs) who were experienced with implementing different methods of screening.

**Method:**

Two independent researchers audio-recorded and analysed interviews using a thematic approach. They asked participants about their experiences with the different methods used for screening AF and which obstacles they faced when implementing screening in daily practice.

**Results:**

In total 15 GPs, nurse practitioners, and HCAs from seven different practices were interviewed. The GP’s office is suited for screening for AF, which ideally should be integrated with standard care. Participants considered pulse palpation, automated sphygmomanometer with AF detection, and single-lead electrocardiography (ECG) as practical tests. Participants trusted pulse palpation over the algorithm of the devices. The follow-up of a positive test with a time-consuming 12-lead ECG hindered integration of screening. The single-lead ECG device reduced the need for immediate follow-up because it can record a rhythm strip. The extra workload of screening and lack of financial coverage form obstacles for implementation.

**Conclusion:**

Pulse palpation, automated blood pressure measure monitors with AF detection, and single-lead ECGs might facilitate screening in a general practice setting. When implementing screening, focus should be on how to avoid disruption of consultation hours by unplanned 12-lead ECGs.

## How this fits in

Pulse palpation, automated sphygmomanometer, and single-lead ECG can detect silent AF in general practice. GPs, nurses, and HCAs found all three methods easy to implement. Clinicians should consider the challenges of how to integrate screening with cardiovascular care and how to manage follow-up of a positive screening.

## Introduction

AF is a common arrhythmia, and prevalence increases strongly with age. Of adults aged >55 years, one in three will be diagnosed with AF during their lifetime.^1^

Some patients with AF experience signs and symptoms such as palpitations, dyspnoea, or lightheadedness.^2^ Other patients are asymptomatic. In a symptomatic patient, the physician will be triggered to check the patient for an arrhythmia. In asymptomatic patients, AF can be detected during routine care if an irregular pulse is noticed; during blood pressure measurement, for example. If a caregiver suspects AF in a patient, a 12-lead ECG or 30-second rhythm strips should be obtained to confirm the diagnosis.^3^

Patients with AF — both symptomatic and asymptomatic — have an increased risk of stroke, heart failure, and other cardiovascular complications.^4,5^ Oral anticoagulant therapy can reduce the risk of stroke risk by more than 60%.^3^ Unfortunately, all too often AF remains undetected and untreated. Previously unknown AF is found in nearly one-quarter of patients who experience stroke.^6^ Identification of unknown ('silent') AF should be a priority as prevention of cardiovascular disease is considered to be an important part of GPs' work. The European Society of Cardiology (ESC) recommends screening for AF in their most recent guideline.^3^

To facilitate detection of AF, new methods have been developed, such as electronic blood pressure monitors with an AF detection function, handheld ECG devices that can record a rhythm strip, and wearable devices. These methods have the potential to facilitate GPs with the identification of silent AF. However, knowledge of how GPs experience screening during everyday practice and which practical issues they face, is lacking. This qualitative study explored how Dutch GPs, nurse practitioners, and HCAs experience screening primary care patients at the GP office, using different methods of screening, and incorporating screening in everyday practice. What hurdles do they experience? What possible factors — such as time, usability, necessary skills — do they consider important when they consider different screening methods?

## Method

### Study design

In this qualitative study, 15 participants were interviewed in seven different practices. Participants consisted of GPs, nurse practitioners, and HCAs). The interviews took place in the GP’s office. All participants were employed in one of the intervention practices of the Detecting and Diagnosing Atrial Fibrillation (D_2_AF) study^7^ and participated in the study. The D_2_AF study investigated opportunistic screening of a random selection of primary care patients, aged ≥65 years. Screening consisted of pulse palpation, an electronic blood pressure monitor with an AF detection function (WatchBP Home A, Microlife, Widnau, Switzerland), and a handheld single-lead ECG device (MyDiagnostick, MyDiagnostick Medical BV, Maastricht, The Netherlands). The single-lead ECG used in the study is a bar with electrodes at both ends, that is able to record a rhythm strip. The strip is immediately analysed after which the bar’s lights indicate 'possible AF' or 'no AF found'. The rhythm strip can only be reviewed after downloading and opening the file on a computer.

Nurses and HCAs often performed the screening under supervision of the GP. Interviews were conducted at the end of the study year.

### Topic list

During the D_2_AF study, participating GPs, nurses, and HCAs provided oral feedback on their experiences. The authors (SU, WL, and HvW) used this feedback to form themes. After discussion among the authors, a topic list was developed that was used for the semi-structured interviews. This list addressed the experiences of the GPs, nurses, and HCAs with the different screening methods, any difficulties to incorporate screening in daily practice, and views on how to implement screening in the future. The list was adjusted when new themes emerged.

### Interviews

A diverse group of practices was included. Practices were approached until data saturation was reached. All seven practices that were approached agreed to participate. One male researcher (SU), a GP in training and trained in qualitative research, performed face-to-face interviews. Interviews took place in the GPs' offices. GPs, nurses, and HCAs were interviewed simultaneously. This setting led to interaction between coworkers within a practice: they explored implications of screening together and commented on each other’s answers. The interviews were audio-recorded and lasted 25–45 minutes.

### Analysis

The audio-recordings were transcribed verbatim, corrected for accuracy, and imported into MAXQDA (version 12). A thematic analysis approach was used because the authors had a general idea of which themes would be important and it allowed for flexibility in the analysis.^8^

Two authors (SU and SB) familiarised themselves with the data by repeated reading of the verbatim transcripts and, if necessary, listening to the original interviews. Then both researchers independently generated initial codes based on the topics. With each interview, the independent analyses were compared and the initial codes were synthesised into one set. After discussion with a third author (WL), the codes were transformed in potential themes. A thematic 'map' of main and sub-themes was developed. During the analyses, themes, sub-themes and their relation was often discussed and refined. Based on the analysis of the first two interviews, the topic list was changed for the subsequent interviews to better understand the importance of false-negative index tests and the role of follow-up testing after the index test. After five interviews, data saturation was reached. In interview six and seven, no new themes were found. When the themes were fully worked out, the report was written out and data were selected with each theme. Supplementary Box S1 shows the final list of themes and sub-themes used in the analysis.

## Results

The researcher (SU) interviewed the participants in seven different practices, ranging from rural to urban, and from solo to group practices. Table 1 shows the characteristics of both the practices and participants. In all interviews, a GP and one or more nurses or HCAs participated. The GPs were a mixed group of males and females of different ages. Participating nurses and HCAs were all female.

**Table 1.** Characteristics of practices and participants (GPs, nurses, and HCAs)

#	Practice location	Type of practice (number of GPs)	GPs' age, years	GPs' sex	GPs' work experience, years	Time GP has worked in current practice, years	Participating HCAs or nurses, *n*	Nurses’ and HCAs' sex
1	Suburban	Solo	41–50	Male	13	4	1	Female
2	Suburban	Group (2)	51–60	Male	24	21	1	Female
3	Rural	Solo	61–70	Male	42	42	1	Female
4	Rural	Solo	51–60	Male	26	25	1	Female
5	Suburban	Solo	41–50	Female	8	3	1	Female
6	Urban	Group (6)	31–40	Male	8	4	2	Female
7	Rural	Group (3)	41–50	Female	16	16	1	Female

HCA = healthcare assistant.

### Logistics

To ensure a safe and private setting for screening, participants expressed that screening should not be done in a public location. A GP’s consultation room could be an appropriate setting because it would ensure proper follow-up. Some believed that the future lies in self-screening by the patient at home, for instance, by using new equipment such as the single-lead ECG device. Some GPs considered screening during flu vaccination, while others dismissed this option because of the already high strain on personnel during these events:

*'* *It should take place in a safe setting;* *n* *ot in a public room, but in the confinement of a consulting room.* *'* (Nurse, practice 1)

*'* *I think that, especially in the future, we’ll see more and more diagnostics take place at home.* *'* (GP, practice 6)

About single-lead ECG: *'Think of our home visits for elderly care.* *You could stick it in your bag and hand it over* [to the patient]*.'* (HCA, practice 4)

An important issue was how to integrate screening with daily practice. Patients eligible for screening need to be selected. Several GPs suggested a built-in function in the electronic medical registration system that reminds caregivers to screen if a patient is at high risk of AF. Furthermore, GPs indicated that they felt able to estimate whether or not screening is warranted, with their knowledge of the patient’s history and personal circumstances. Most physicians prefer to ingrate screening with standard care, such as a routine cardiovascular check-up:

'*If you can combine it with other preventive care* […] *that saves time*.' (GP, practice 1)

*'* *Combining it with cardiovascular care, or checking blood pressure* […] *I do not think we check the pulse by hand in every cardiovascular check-up? Maybe we should do this.* *'* (GP, practice 5)

Most participants do not prefer integration of screening into a regular consultation because of the organisational issues involved: first, the time it takes to explain why patients are selected for screening and the importance of it; second, the efforts of the initial screening; and third, the follow-up required if the index test is positive. The last argument was emphasised because follow-up of a positive index test with an ECG is time-consuming and cannot be planned in advance, so can lead to an additional appointment at a later time:

Interviewer: And using the single-lead ECG in the consultation room, would that disrupt the workflow? *'Yes it would* […] *It takes time. You have to explain why you are doing it.' (*GP, practice 6)

GP to practice nurse: *'* *For me the issue is that, the few times we find something, it does not mess up your consultation hours* […] *The point is, if the test is good, then I’m done. But if it is not good, than I have to take action. That takes up my clinic time.* *'* (GP, practice 1)

### Testing methods and characteristics

In general, participants considered all three methods easy to use. Pulse palpation is a quick and readily at hand method of screening. The physical contact can also provide comfort to a patient. The participants thought that the use of equipment with AF detection raised their awareness for silent AF. The single-lead ECG required some preparation in advance, but use of it was intuitive after that. The single-lead recording can be stored and interpreted at a later time. If the physician can identify a clear cause for an irregular rhythm on a positive test, no follow-up with a 12-lead ECG is necessary. This reduces the need for immediate follow-up with a 12-lead ECG. The automated sphygmomanometer takes three blood pressure measurements, which takes some time. This was not found troublesome by most participants because it allowed them to complete other tasks during the measurement. However, some GPs preferred manual blood pressure measurement for speed. In practices the sphygmomanometer was used as part of regular care more often than the single-lead ECG:

On pulse palpation: *'* *If you also give some soothing contact, it provides relaxation and a better understanding of the patient.* *'* (Nurse, practice 2)

*'* *I think equipment will improve our awareness.* *'* (GP, practice 5)

'*Making a* [12-lead] *ECG, it takes 7 minutes or so* […] *The single-lead ECG takes one minute and you can check: is it atrial fibrillation?* […] *Not having to make an 12-lead ECG, would be practical.*' (GP, practice 5)

GPs trusted their ability to differentiate between an extra systole and AF using pulse palpation and auscultation. Nurses and HCAs often consulted with the GP in case of an irregular pulse. Trust in the devices was less obvious. Both devices use a light indicating that the built-in algorithm has detected an irregularity. The single-lead ECG produces a rhythm strip, which is only visible after the user has downloaded this on a computer. Participants indicated that they found it difficult to let their actions be based on the outcome of the indicator light without additional visual feedback. Participants had the impulse to confirm a positive result on the device by pulse palpation and/or auscultation. With increasing experience, the trust in the devices grew.

Participants preferred a test with a high specificity over high sensitivity because with high specificity fewer patients are falsely selected for follow-up with an ECG. This is especially important when screening for a low-prevalence condition such as silent AF, which leads to many false positive tests. Missing a case of AF was acceptable for GPs:

'*I think that in the case of a single extra beat* *—* *I would prolong pulse palpation for a short while, but not necessarily make an ECG* […] *If the extra beat is only sporadic, I would leave it at that.*' (GP, practice 1)

*'I felt a very distinct extra beat and an irregular rhythm. I thought, something is wrong. However, it turned out to be fine. The single-lead ECG had shown a green light* [no irregularity found]. *Can you rely on this?' (*Nurse practitioner, practice 2)

*'* *If you could* […] *lower the number of times follow-up is needed, that would decrease the burden a lot.* *'* (GP, practice 1)

On missing a case: *'* *No, that’s the risk of screening, but at least you have tried.* *'* (GP, practice 1)

### Financial compensation

Some GPs indicated that for a structured screening system to be organised in addition to regular care, an additional time investment is needed of the personnel. The screening process is time-consuming and most GPs indicated that they would delegate the task to their staff. This will require additional funding:

*'* *If I delegate a task* [to a nurse or HCA]*, it has to be reimbursed.* *'* (GP, practice 4)

## Discussion

### Summary

Screening for AF should be done in a GP's office (or in the future at home), and should be integrated with regular care, for instance, with a cardiovascular check-up. Participants thought it was feasible to implement pulse palpation, automated sphygmomanometer, and single-lead ECGs in daily practice. Participants indicated that the presence of equipment with a function to detect AF in the office, raised awareness for silent AF. GPs, nurses, and HCAs said they had a tendency to check a positive screening by the devices with pulse palpation. The follow-up of a positive test with a 12-lead ECG seemed to hamper integration of screening. The single-lead ECG recording solves this problem because it reduces the number of cases that need follow-up of a positive test with a 12-lead ECG.

### Strength and limitations

A selected group of GPs, nurses, and HCAs were interviewed. The participants took part in the trial on screening for AF because of their experience with three different methods of screening. Their willingness to take part in this trial probably means that the participants already had a specific motivating interest in cardiovascular care. Furthermore, only one GP from each practice was interviewed.

The GPs, nurses, and HCAs were interviewed simultaneously in their own practice. A disadvantage of this method is that participants might have influenced each other, for instance, owing to a hierarchical relationship between GPs and coworkers. However, an open atmosphere was experienced during the interviews, and GPs, nurses, and HCAs were seen discussing their experiences and exploring implications of screening for their practice, without apparent restraint.

### Comparison with existing literature

The Dutch College of General Practitioners guideline for AF recommends assessing heart rhythm in symptomatic patients and during blood pressure measurement, without specifying a preferred method.^9^ It does not recommend screening. The ESC recommends using pulse palpation or using a single-lead ECG device for opportunistic screening.^3^ The National Institute for Health and Care Excellence (NICE) guideline recommends the use of automated sphygmomanometers with AF detection.^10^ A Dutch vignette study showed that one in five GPs is already using these devices.^11^ The use of equipment with an AF-detection function raised awareness of silent AF, something that Orchard *et al* also confirmed in a qualitative study among Australian GPs.^12^ All methods have a high negative predictive value and a low positive predictive value when used in screening for AF.^3^ This means that only a positive screening needs follow-up with an ECG registration.

The study showed that for GPs, pulse palpation has the advantages of ease of use and of having no additional costs. Furthermore, some healthcare professionals consider the physical contact between professional and patient using pulse palpation as valuable. Taggar *et al* showed that GPs and non-GP healthcare professionals in the UK regularly perform pulse palpation, although GPs are more confident in identifying an irregular pulse than non-GP healthcare professionals.^13^ Participants often also trusted their own observation more than the result of the device’s algorithm. Once caregivers become familiar with the devices, this effect seems to disappear.

The single-lead ECG used in the study records a rhythm strip that can be viewed at a later time. Visualisation of the single-lead ECG produced by the device may also increase acceptance of the result of the algorithm. Orchard *et al* interviewed Australian GPs and nurses who had screened patients using a single-lead ECG for screening with a real-time trace on a smartphone screen.^14^ The majority found screening with the device engaging and were confident in using the equipment. A minority reported experiencing technical difficulties. Lowres *et al* interviewed Australian pharmacists experienced with screening customers using a single-lead ECG with smartphone registration.^15^ The new technology drew interest of the customers and increased awareness of their own heart rhythm.

The single-lead ECG can identify common causes for an irregular heartbeat, such as multiple ventricular extra systoles. If a clear cause for irregularity is found when reviewing the single-lead ECG and no AF is detected, no follow-up with a time-consuming 12-lead ECG is necessary. The study showed that healthcare workers viewed such disruption of consultations negatively. On the other hand, if the likelihood of detecting AF is high, GPs regard screening as worthwhile. A test with fewer false positive results would reduce the number of unnecessary 12-lead ECGs. Pulse palpation has a sensitivity of 92% and a specificity of 82%.^16^ Assuming a prevalence of 1.44%,^17^ the low positive predictive value of 7% would lead to 14 negative ECGs for every case of AF. Electronic blood pressure monitors with an AF detection function and single-lead ECGs have a higher sensitivity and specificity of up to 92–98% and 92–95%, respectively.^16,18,19^ Using one of these methods reduces the number of required 12-lead ECGs by half. Interestingly, the findings showed that for most GPs this reduction of the workload of screening was more important than increasing the sensitivity of screening, prioritising logistics over yield of screening. Most Dutch GPs feel competent to diagnose AF, and either have a 12-lead ECG on site or are able to refer patients to an external facility without consulting a specialist.^11^ Often the HCA or nurse is responsible for conducting ECGs.^13^ The participating practices all had established cardiovascular care programmes. Furthermore, Dutch GPs have a central role in the healthcare system and a thorough knowledge of the patient’s history, which makes the GP’s office a suitable place for screening. GPs in other countries may work in different circumstances and have different views on screening for AF than those represented in this study.

Some GPs indicated that the additional workload and strain on employees should be compensated for. This is in line with the findings of a survey in the UK, which stressed that the workload and available funding are possible barriers for screening in current practice.^13^

### Implications for research and practice

Future research on screening for AF should not only focus on the efficacy of an intervention, but also should consider how to integrate the screening process within daily practice. Pulse palpation, automated blood pressure measure monitors with AF detection, and single-lead ECGs all have their own merits, and GPs, nurses, and HCAs regarded all methods easy to use. An important consideration should be how to prevent disruption of consultation hours by unplanned 12-lead ECGs. GPs who intend to start screening should plan in advance how best to mitigate this issue.
